# Mechanism of QSYQ on anti-apoptosis mediated by different subtypes of cyclooxygenase in AMI induced heart failure rats

**DOI:** 10.1186/s12906-015-0869-z

**Published:** 2015-10-07

**Authors:** Jing Wang, Chun Li, Yuan Cao, Qiyan Wang, Linghui Lu, Hong Chang, Yan Wu, Jing Han, Wei Wang, Pengfei Tu, Yong Wang

**Affiliations:** Modern Research Center for Traditional Chinese Medicine, School of Chinese Materia Medica, Beijing University of Chinese Medicine, Beijing, China; Basic Medical College, Beijing University of Chinese Medicine, Beijing, China; Scientific Research Centre, Beijing University of Chinese Medicine, Beijing, China

## Abstract

**Background:**

Qi-shen-yi-qi (QSYQ), one of the most well-known traditional Chinese medicine (TCM) formulas, has been shown to have cardioprotective effects in rats with heart failure (HF) induced by acute myocardial infarction (AMI). However, the mechanisms of its therapeutic effects remain unclear. In this study, we aim to explore the mechanisms of QSYQ in preventing left ventricular remodelling in rats with HF. The anti-apoptosis an anti-inflammation effects of QSYQ were investigated.

**Methods:**

Sprague–Dawley (SD) rats were randomly divided into 4 groups: sham group, model group, QSYQ treatment group and aspirin group. Heart failure model was induced by ligation of left anterior descending (LAD) coronary artery. 28 days after surgery, hemodynamics were detected. Echocardiography was adopted to evaluate heart function. TUNEL assay was applied to assess myocardial apoptosis rates. Protein expressions of cyclooxygenase1 and 2 (COX1and COX2), Fas ligand (FasL), P53 and MDM2 were measured by western-blot. RT-PCR was applied to detect expressions of our subtype receptors of PGE2 (EP1, 2, 3, and 4).

**Results:**

Ultrasonography showed that EF and FS values decreased significantly and abnormal hemodynamic alterations were observed in model group compared to sham group. These indications illustrated that HF models were successfully induced. Levels of inflammatory cytokines (TNF-α and IL-6) in myocardial tissue were up-regulated in the model group as compared to those in sham group. Western-blot analysis showed that cyclooxygenase 2, which is highly inducible by inflammatory cytokines, increased significantly. Moreover, RT-PCR showed that expressions of EP2 and EP4, which are the receptors of PGE2, were also up-regulated. Increased expressions of apoptotic pathway factors, including P53 and FasL, might be induced by the binding of PGE2 with EP2/4. MDM2, the inhibitor of P53, decreased in model group. TUNEL results manifested that apoptosis rates of myocardial cells increased in the model group. After treatment with QSYQ, expressions of inflammatory factors, including TNF-α, IL-6 and COX2, were reduced. Expressions of EP2 and EP4 receptors also decreased, suggesting that PGE2-mediated apoptosis was inhibited by QSYQ. MDM2 was up-regulated and P53 and FasL in the apoptotic pathway were down-regulated. Apoptosis rates in myocardial tissue in the QSYQ group decreased compared with those in the model group.

**Conclusions:**

QSYQ exerts cardiac protective efficacy mainly through inhibiting the inflammatory response and down-regulating apoptosis. The anti-inflammatory and anti-apoptosis efficacies of QSYQ are probably achieved by inhibition of COXs-induced P53/FasL pathway. These findings provide experimental evidence for the beneficial effects of QSYQ in the clinical application for treating patients with HF.

**Electronic supplementary material:**

The online version of this article (doi:10.1186/s12906-015-0869-z) contains supplementary material, which is available to authorized users.

## Background

Heart failure (HF) induced by acute myocardial infarction (AMI) remains the leading cause of morbidity and mortality worldwide, inspite of extensive investigations [1]. Exploration of effective prevention and therapy for HF poses a major challenge to the entire medical community. The pathogenesis of HF and new therapeutic approaches for HF need to be investigated further.

Abundant evidence indicates that inflammation and apoptosis play important roles in the development of HF [2–4]. Previous studies found that inflammatory cytokines promote development of HF [5, 6]. In particular, arachidonic acid (AA) metabolism plays an important role in HF development [7, 8]. The key rate-limiting enzymes in AA pathway are cyclooxygenases (COXs) and they have been used as targets of non-steroidal anti-inflammatory drugs (NSAIDs) in clinical treatment of HF. Large-scale randomized clinical experiments showed that aspirin, along with other NSAIDs which target COXs, has cardio-protective effects [9]. COX1 and COX2 are the two isoenzymes of cyclooxygenases. COX1 is expressed constitutively in most tissues, whereas COX2 is the inducible form of the enzyme that is produced upon stimulation by growth factors and cytokines (e.g., inflammation) [10].

Myocardial apoptosis has been identified as another essential process in the development of HF [11]. Activation of apoptotic pathways leads to myocyte damage and eventual myocardial fibrosis. P53-dependent myocardial apoptosis is one of the apoptotic pathways that contribute to progress of HF [12]. P53 activates the extrinsic apoptotic pathway by triggering the expression of transmembrane protein FasL, whose receptor belongs to TNF receptor family (TNF-R) [13, 14]. The activation of that particular ‘death’ receptor (TNF-R) family leads to a cascade expression of caspases, including caspase-8 and caspase-3, which in turn enhances apoptosis [15]. Overexpression of the FasL antigen has been reported in myocardial infarction tissues in rats [16]. As activation of P53 pathway can lead to many significant outcomes, the expression of P53 needs to be strictly regulated. MDM2 could bind with P53 gene and down-regulate P53 level by inhibiting its transcriptional activity [17]. Numerous studies have demonstrated that AA and its metabolic intermediates can lead to apoptosis. For example, Prostaglandin E2 (PGE2) is a downstream metabolite of the AA pathway and could induce apoptosis in a dose-dependent manner [18, 19]. Specifically, PGE2 can increase P53 transcriptional activity by binding with the subtype receptor EP2, thereby inducing apoptosis [20]. Activation of EP2/EP4 receptors by PGE2 can also promote apoptosis by increasing expressions of FasL, caspase8 and caspase9 [21]. The AA pathway also exerts diverse effects on the inflammation process through promoting the mRNA expression of receptor activator of NF-κB [22]. Therefore, factors in the AA pathway would be ideal potential targets for the treatment of HF [23, 24]. A series of experimental studies have been carried out to investigate treatment of HF by inhibiting AA metabolism, specifically by suppressing activation of COXs. However, traditional NSAIDs (such as aspirin) have non-specific inhibition effects on COX1 and COX2, resulting in side effects such as gastrointestinal diseases [25, 26]. On the other hand, specific COX2 inhibitors increase the risk of cardiovascular diseases [27].

Traditional Chinese medicines (TCM) have been used in the treatment of HF for thousands of years, and many drugs have been proven effective [28]. QiShenYiQi (QSYQ) was a patent prescription of TCM approved by China’s Food and Drug Administration (CFDA) in 2003 for treating HF (Approval number of CFDA: Z20030139) [29]. Briefly, QSYQ comprises four medical herbs, namely Radix Salvia miltiorrrhiza (Danshen), Panax notoginseng (Sanqi), Radix Astragalus membranaceus (Huangqi), and Dalbergia odorifera T. Chen (Jiangxiang). The major active ingredients are ginsenosides Rg1 and Rb1 (from Sanqi), astragaloside (from Huanqi), and tanshinol (from Danshen) [30, 31]. Large-scale randomized and controlled clinical trials have proven that QSYQ has a definite effect in improving heart function [32, 33]. Animal studies also demonstrated that QSYQ could effectively attenuate myocardial fibrosis, probably through inhibiting the renin-angiotensin-aldosterone pathway [34]. Moreover, studies showed that QSYQ has an anti-inflammatory effect in rats with AMI-induced HF [35]. Our previous study confirmed that QSYQ can attenuate inflammation by inhibiting STAT3 and NF-κB signalling pathway [36]. Recently, network Pharmacology has been applied to reveal the underlying pharmacological mechanisms of QSYQ. Apoptosis was predicted to be one of the most likely targeted pathways of QSYQ [37]. However, whether the efficacy of QSYQ is exerted by direct inhibition of myocardial apoptosis or anti-inflammatory response remains to be validated.

In this study, we aim to investigate the underlying pharmacological mechanisms of QSYQ in HF model. The effects of QSYQ on AA pathways and COXs-P53-FasL mediated apoptosis pathway were studied. HF model was induced by ligation of the left anterior descending (LAD) coronary artery in rats. This study will provide insights into the clinical treatment of HF induced by AMI.

## Methods

### Animals and grouping

Studies were performed according to the “Guide for the Care and Use of Laboratory Animals” published by National Institutes of Health (NIH Publications No. 85–23, revised 1996) and with approval of the Animal Care Committee of Beijing University of Chinese Medicine. A total of 60 male Sprague–Dawley (SD) rats with weights of 240 g ± 10 g in SPF grade were selected (purchased from Beijing Vital River Laboratory Animal Technology Co. Ltd.).

### HF model preparation and study designs

**Fig. 1:**
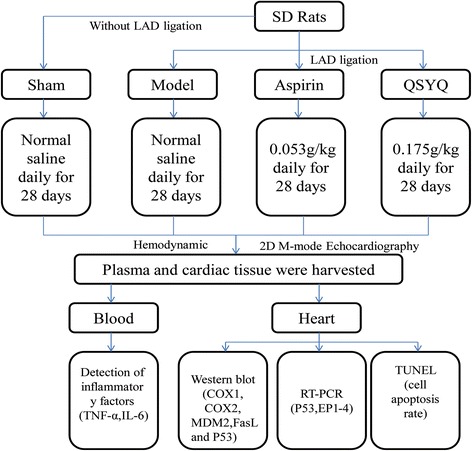
Study designs. Left anterior descending (LAD) coronary artery was ligated to induce the heart failure (HF) model. Rats in the sham groups went through identical procedures except that their LAD arteries were not ligated. The rats alive after surgery were randomly divided into three groups, with ten rats in each group. There were also ten rats in the sham groups. QSYQ dissolved in water was given by oral gavage for 28 days at a total dosage of 0.175 g/kg on a daily base. Aspirin was given as a control drug at a dosage of 53.3 mg/kg

HF was induced by direct left anterior descending (LAD) ligation as described previously [34]. Briefly, SD rats were anaesthetized with 1 % pentobarbital sodium (50 mg/kg ip), and a left thoracotomy was performed. The LAD was ligated near its origin at the edge of the left atrium (Surgipro, CT, USA). The thorax was then closed. The rats were kept on a heating pad and monitored until the sedation wore off and they were awake. Live rats were then randomly divided into three groups: ten in the model group, 10 in the Aspirin group, and ten in the QSYQ group. Meanwhile, the ten rats in the sham-operated group without LAD ligation were investigated together. The QSYQ group was treated for 28 days by daily oral gavage of QSYQ with a total daily dose of 0.175 g/kg (Tasly Pharmaceutical Group Corporation. Tianjin, China, Series: 100706). QSYQ was dissolved in water. The fingerprints of QSYQ established by the HPLC-IT-TOF method were shown in Additional file 1. The sham-operated group and the model group received the same volume of water, and the Aspirin group was given the same volume of Aspirin solution (53.3 mg/kg) via oral gavage. The study designs were carried out as shown in Fig. 1.

The overall mortality rate of HF rats during the entire experimental period (up to 28 days after MI) was 30 to 40 %. The majority of death occurred on the day of or the day after the MI surgery, probably due to acute pump failure or lethal arrhythmias.

### Hemodynamic measurements in rats

Left ventricular (LV) performance was measured in rats anesthetized with 2 % isoflurane as previously described [36]. A terminal hemodynamic study was performed at 28 days after surgery. Right carotid artery, left ventricular systolic and end-diastolic, diastolic and mean aortic pressures, Max dP/dt, Min dP/dt, and heart rate were recorded by using a system of PowerLab ML880 (AD Instrument, Australia).

### Echocardiographic assessment of left ventricular function

Echocardiography was applied to detect the left ventricular end-systolic diameter (LVESd), left ventricular end-diastolic diameter (LVEDd), left ventricular end- diastolic volume (LVEDV), left ventricular end-systolic volume (LVESV), ejection fraction (EF), fractional shortening (FS), and other indicators. A PST 65A sector scanner (8-MHz probe) was employed, which generates two-dimensional images at a frame rate of 300 to 500 frames/s. The LV dimension (LVD) was measured using M-model ractional shortening, and FS% was calculated using the following equation: FS % = [(LVEDd ‐ LVESd)/LVEDd] × 100. EF % was calculated using the following equation: EF % = [(LVEDV ‐ LVESV)/LVEDV] × 100 %.

### Terminal deoxynucleotidyl transferase dUTP Nick end labeling (TUNEL)

The cell apoptosis rate was determined by TUNEL according to manufacturer’s instructions (Roche Applied Science, South San Francisco, CA, USA). Four micrographs were randomly selected. To assess the fraction of apoptotic cells, the count of TUNEL-positive cells were divided to do ratio with the total number of hematoxylin-positive cardiac myocyte nuclei.

### Measurement of serum indicators by ELISA

Levels of TNF-α and IL-6 were quantified by commercial ELISA kits (Crystal Chem Inc., Downer’s Grove, USA) and levels of PGE2 were quantified by Prostaglandin E2 Assay of R&D Systems. Each assay was performed following respective instructions. Standards at a series of concentrations were run in parallel with the samples. The concentrations in the samples were calculated in reference to the corresponding standard curves and were expressed as pg/mL.

### Measurement of indicators by western blot

The cardiac tissue was homogenized in RIPA buffer (50 mM Tris–HCl Ph7.4, 150 mM NaCl, 1 % NP-40, 0.1%SDS) and total protein was extracted from this homogenate. The protein concentration in each sample extract was measured by a protein assay kit (Beijing PuLilai Gene Technology Co., Ltd, Beijing, China, lot number: P1511) and was then adjusted to the same value in all samples with 5X SDS-PAGE sample buffer. The samples were boiled for 5 min followed by loading on a 10 % SDS-PAGE gel (50ug protein/10uL per well) for electrophoresis using a Bio-Rad mini gel apparatus at 100 V for 2 h. The fractionated protein on the gel was transferred onto a NC membrane (Beijing PuLilai Gene Technology Co., Ltd, Beijing, China, lot number: P2110) and electrophoresed at 300 mA for 90 mins. The membrane was first probed with COX1 and secondary antibody (goat polyclonal secondary antibody to rabbit IgG-HRP, ab97064, Abcam, 1:500), and then treated with ECL (ECL Plus western blotting detection reagent, GE Healthcare) for 1 min at room temperature. The bands in the membrane were visualized and analyzed by Imagelab software. After obtaining the COX1 blot density, the COX2, FasL, MDM2, GAPDH blot densities were obtained by the same procedures (primary antibodies of COX1, COX2, FasL, MDM2, P53 are as follows. Rabbit monoclonal to COX1, Abcam, USA. Ab109025; Rabbit monoclonal to COX2, Abcam, USA, Ab15191; Rabbit polyclonal to FasL, Abcam, USA, Ab82419; Rabbit polyclonal to MDM2, Abcam, USA, Ab38618; Rabbit polyclonal to P53, Abcam, USA, Ab131442; GAPDH mouse monoclonal IgG, Ab8245, Abcam, 1:2000). The final reported data were normalized by GAPDH.

### Measurement of P53 and EPs expression by real-time PCR

The mRNA expressions of P53, EP1, EP2, EP3 and EP4 were determined by reverse transcriptase polymerase chain reaction (RT-PCR). Total RNA was extracted using TRIzol Reagent (Gibco-BRL, Paisley, UK). The concentration of RNA was measured using Nano Drop 2000 (Thermo Scientific, USA), and then the RNA was transcribed using the RevertAidTM First Stand cDNA Synthesis kit (Fermentas, LT). The reaction volume was 20ul and it contained 2ul cDNA, 0.5ul forward and reverse primers, 10ul Rox and 7ul DEPC. The reactions were carried out for 40 cycles. For cDNAs analysis of P53, EP1-4 and GAPDH, PCR conditions were set as follows: 15 s at 95 °C for denaturation, and 1 min at 55 °C for annealing and extension. The sequences of the primers were shown at Table 1. Quantities of P53, EP1, EP2, EP3 and EP4 mRNA were normalized to GAPDH mRNA level. The relative quantitative expressions of these genes were calculated by the formula “ gene expression = 2^ΔCT (ΔCT = GAPDH CT ‐ gene of interest CT)^ ”.

**Table 1:** Nucleotide sequences of primers used in real-time PCR

Gene (accession no.)	Primes	Nucleotiede sequences5‘-3’	Size(bp)	Temp(°C)
EP1	Forward	CTGCGTCATCCATCACTT	165	55.0
	Reverse	CCAACACCACCAATACCA		55.0
EP2	Forward	CGGATTGTCTGGCAGTAG	124	57.3
	Reverse	GCATACAGCGAAGGTGAT		55.0
EP3	Forward	CTTCAATCAGATGTCAGTAGAG	181	57.8
	Reverse	CCGCTTCAGGTTGTTCAT		55.4
EP4	Forward	CTATACCTGCCAGACCTAAC	174	56.3
	Reverse	CTCATCCACCAACAAGACA		57.3
GAPDH	Forward	TTCAACGGCACAGTCAAG	116	55.0
	Reverse	TACTCAGCACCAGCATCA		55.0

### Statistical analysis

All data were presented as mean ± standard deviation (SD). Statistical analysis was performed with the SPSS program package (SPSS version 20.0) or GraphPad Prism 5. Statistical analysis was carried out using one-way analysis of variance (ANOVA) and Tukey’s test. The values of *P* < 0.05 were considered as statistically significant.

## Results

### QSYQ restored LAD-induced hemodynamic alterations

**Fig. 2:**
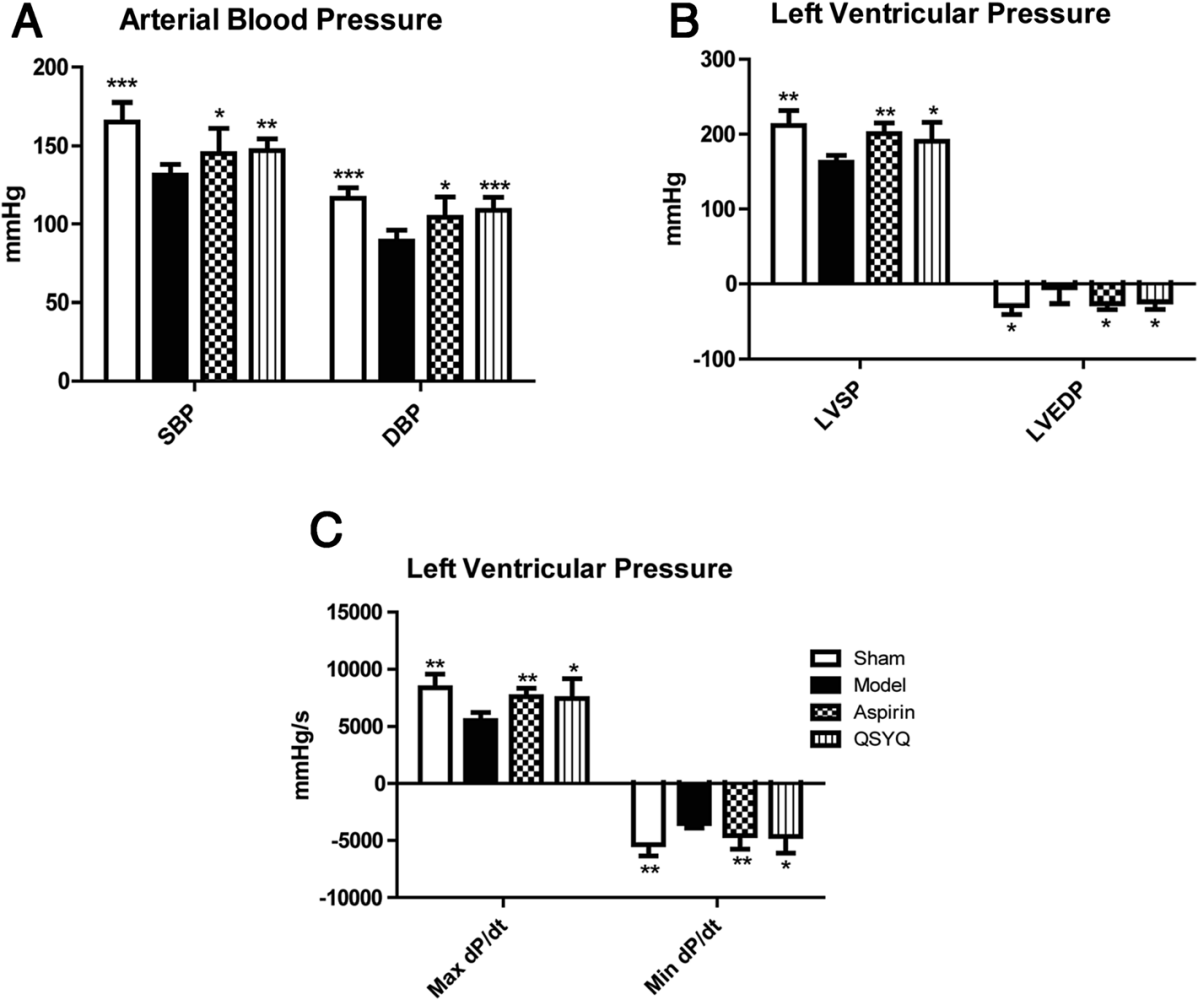
QSYQ Improved hemodynamic alterations. **a** Values of SBP and DBP, which reflect arterial blood pressures, were reduced in the model group. QSYQ and aspirin increased SBP and DBP towards normal levels; (**b**) and (**c**) Values of LVSP, LVEDP, Max dp/dt and Min dp/dt, which reflect the left ventricular pressures, were down-regulated in the model group. QSYQ up-regulated LVSP, LVEDP, Max dp/dt and Min dp/dt, suggesting that QSYQ can regulate hemodynamic alterations induced by ischemia (*P < 0.05, **P < 0.01, ***P < 0.001, other groups vs. model group, *n* = 8)

Diastolic and systolic LV functions were impaired in the HF model group, whereas those in the sham group were intact. LV functions were significantly improved after treatment with QSYQ for 28 days. The values of systolic blood pressure (SBP) and diastolic blood pressure (DBP) significantly increased in QSYQ group, as compared to those in the model group (*P* < 0.001). The values of LV systolic pressure (LVSP) and Max dP/dt, which reflect LV systolic function, were also restored to basal levels after treatment with QSYQ (*P* < 0.05). Compared to the sham group, LV end-diastolic pressures (LVEDP) and Min dP/dt values were up-regulated in the model group, suggesting an impaired diastolic function in the model rats. QSYQ could restore diastolic function by reducing values of LVEDP and Min dP/dt in rats with HF (*P* < 0.05). The efficacy of aspirin was similar with that of QSYQ, as shown in Fig. 2.

### QSYQ improved cardiac function

**Fig. 3:**
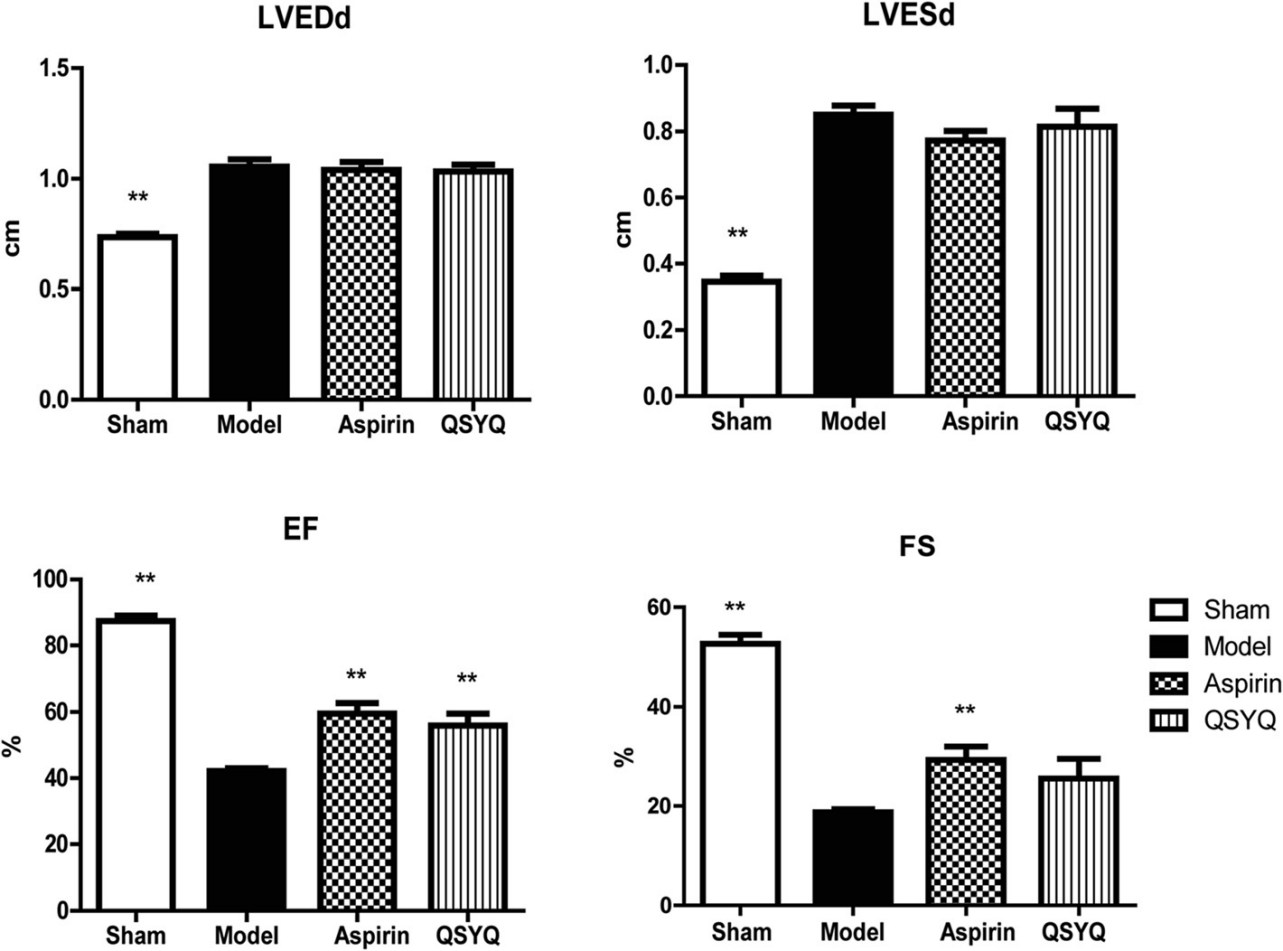
QXYQ improved cardiac function. Parameters of cardiac function were detected by echocardiography. LVEDd and LVESd were up-regulated and EF and FS were reduced in the model group compared with those in the sham group. QSYQ didn’t show effect on LVEDd. LVESd was reduced to a certain degree in QSYQ group compared with that in model group (*P* > 0.05). EF and FS were up-regulated in QSYQ group compared with those in model group, suggesting that QYSQ could improve cardiac function (*P < 0.05, **P < 0.01, other groups vs. model group, *n* = 8)

28 days after surgery, echocardiography showed that values of EF and FS were significantly reduced in the model group compared with those in the sham group. An increase in values of LVEDd and LVESd was also observed in the model group, suggesting that cardiac hypertrophy was induced in this stage (the primary data were listed in Additional file 2). After treatment with QSYQ for 28 days, LVEDd was not affected but LVESd was reduced to a certain degree (*P* > 0.05). Reduction of LVESd/LVEDd ratio resulted in an increase of EF and FS values in QSYQ group compared with those in the sham group (Fig. 3).

### QSYQ inhibited apoptosis in AMI-induced HF

**Fig. 4:**
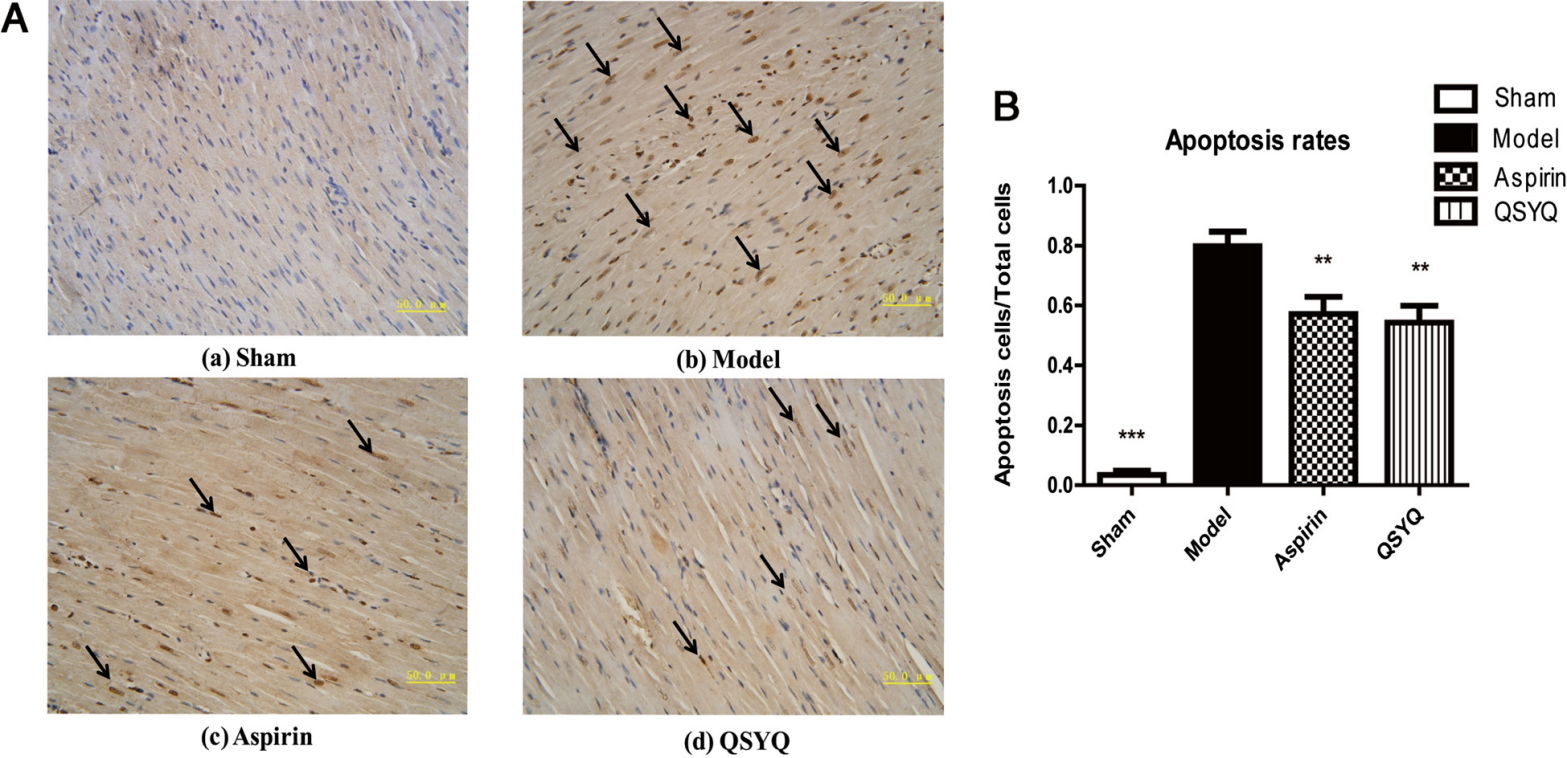
QSYQ inhibited cell apoptosis in the rats with HF. **a** TUNEL analysis was carried out 28 days after surgery and drug treatments. Figure (**a**) showed that almost all the cells in the sham group were TUNEL- negative cells (living cells); Figure (**b**) showed that there were a number of TUNEL-positive cells (apoptotic cells indicated by arrows) in the model group; Figure (**c**) and (**d**) showed that numbers of apoptotic cells were reduced in aspirin and QSYQ groups compared with those in the model group. **b** The percentages of apoptotic cells in heart tissues were quantified in four groups of rats. QSYQ reduced cardiac apoptosis rate in the HF rats and aspirin could exert a similar effect (**P < 0.01, ***P < 0.001, other groups vs. model group, *n* = 8)

Myocardial apoptosis is one of the major pathological changes of HF. TUNEL assay illustrated that numbers of apoptotic myocardial cells in the HF group were significantly higher than those in the sham group, whereas QSYQ treatment dramatically reduced apoptosis rates (Fig. 4). Aspirin also showed an anti-apoptosis effect, as has been reported before [38].

**Fig. 5:**
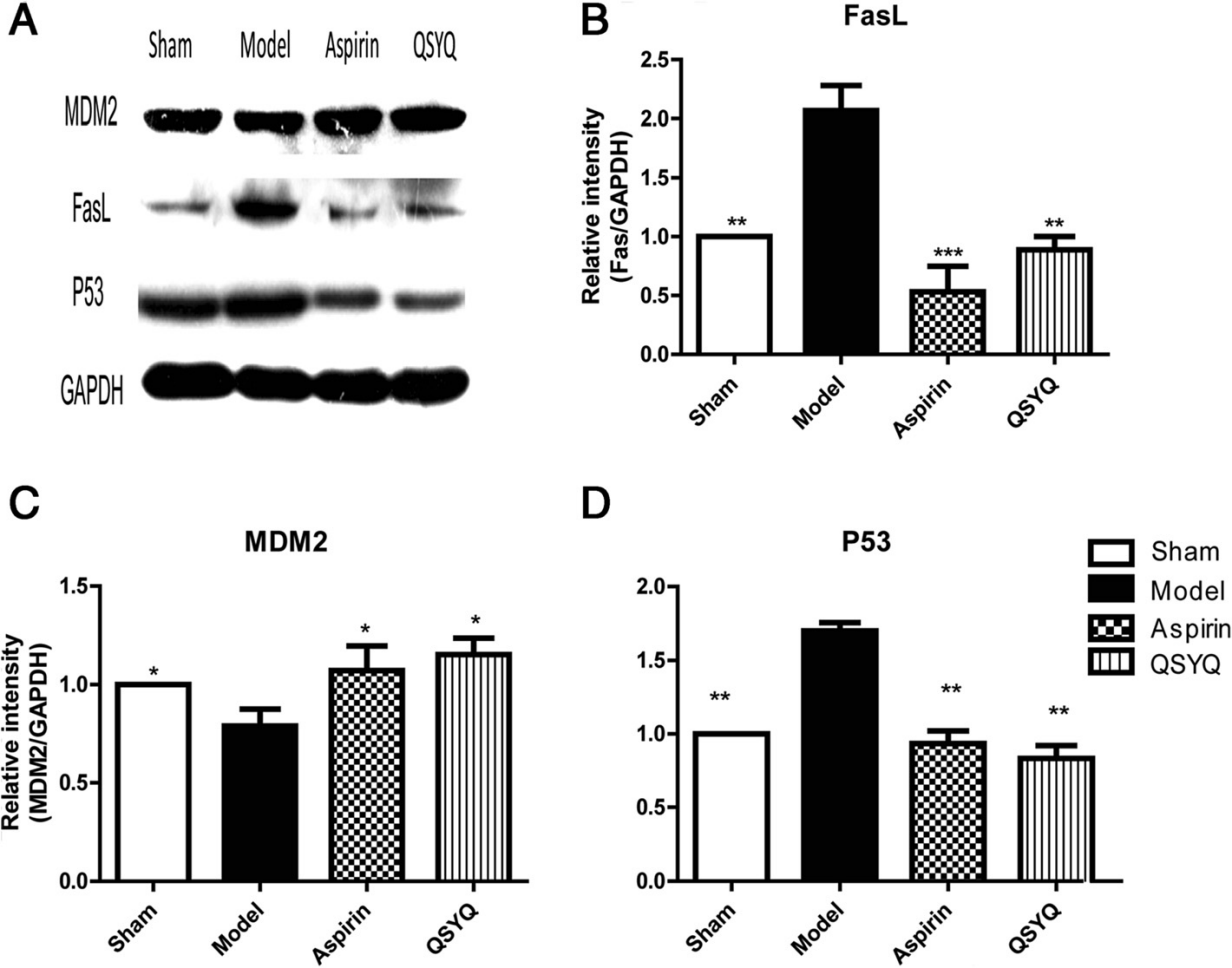
QSYQ attenuated cardiac apoptosis. **a** Western blot showed that expression of MDM2 was down-regulated and expressions of FasL and P53 were up-regulated in the model group compared with those in the sham group. After treatments with aspirin and QSYQ, expression of MDM2 was elevated and expressions of FasL and P53 were reduced, as compared with those in model group. Figure (**b**), (**c**) and (**d**) were relative quantitative illustrations of expressions of FasL, MDM2 and P53 in the four groups of rats (*P < 0.05, **P < 0.01, ***P < 0.001, other groups vs. model group, *n* = 8)

Over-expressions of P53 and FasL are indicators of apoptosis activation. Western-blot analysis showed that expressions of FasL and P53 were significantly up-regulated in the model group compared with those in the sham group. MDM2 is an inhibitor of P53 transcription. Expression of MDM2 was down-regulated in the model group compared with that in the sham group (Fig. 5). Combined with the TUNEL experiment, these results indicated that apoptosis was activated in rats with HF. QSYQ greatly suppressed apoptosis by activating MDM2 expression and inhibiting expressions of FasL and P53 (Fig. 5). Altogether, these results demonstrated that QSYQ has an anti-apoptosis effect in rats with HF.

### QSYQ attenuated inflammation and inhibited COXs

**Fig. 6:**
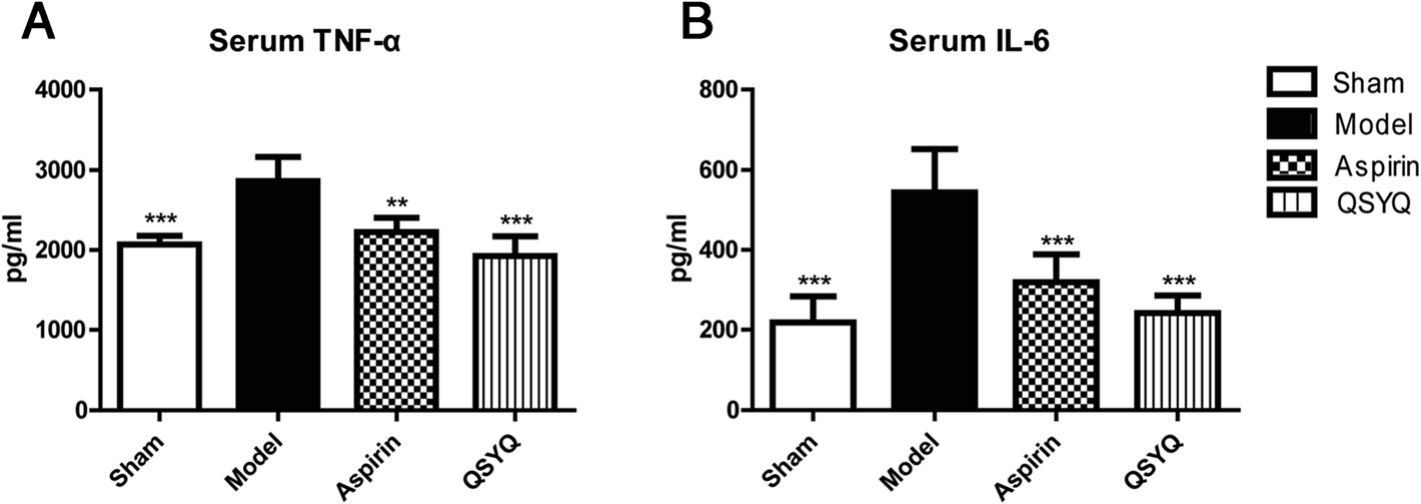
QSYQ attenuated inflammation. **a** ELISA was applied to detect the serum levels of TNF-ɑ in different groups. **b** The serum levels of IL-6 in different groups were also detected by ELISA. The results showed that in the model group, the levels of TNF-ɑ and IL-6 significantly increased compared with those in the sham group. In aspirin and QSYQ group, the levels of TNF-ɑ and IL-6 decreased as compared with those in the model group (**P < 0.01, ***P < 0.001, other groups vs. model group, *n* = 8)

TNF-α and IL-6 are important inflammatory cytokines which mediate inflammation. ELISA results showed that expressions of TNF-α and IL-6 were up-regulated in model group compared with those in the sham group (Fig. 6). Levels of TNF-α and IL-6 were down-regulated in the QSYQ treatment group compared with those in the model group, indicating an anti-inflammatory effect of QSYQ. Aspirin also reduced expressions of TNF-α and IL-6 (*P* < 0.01).

**Fig. 7:**
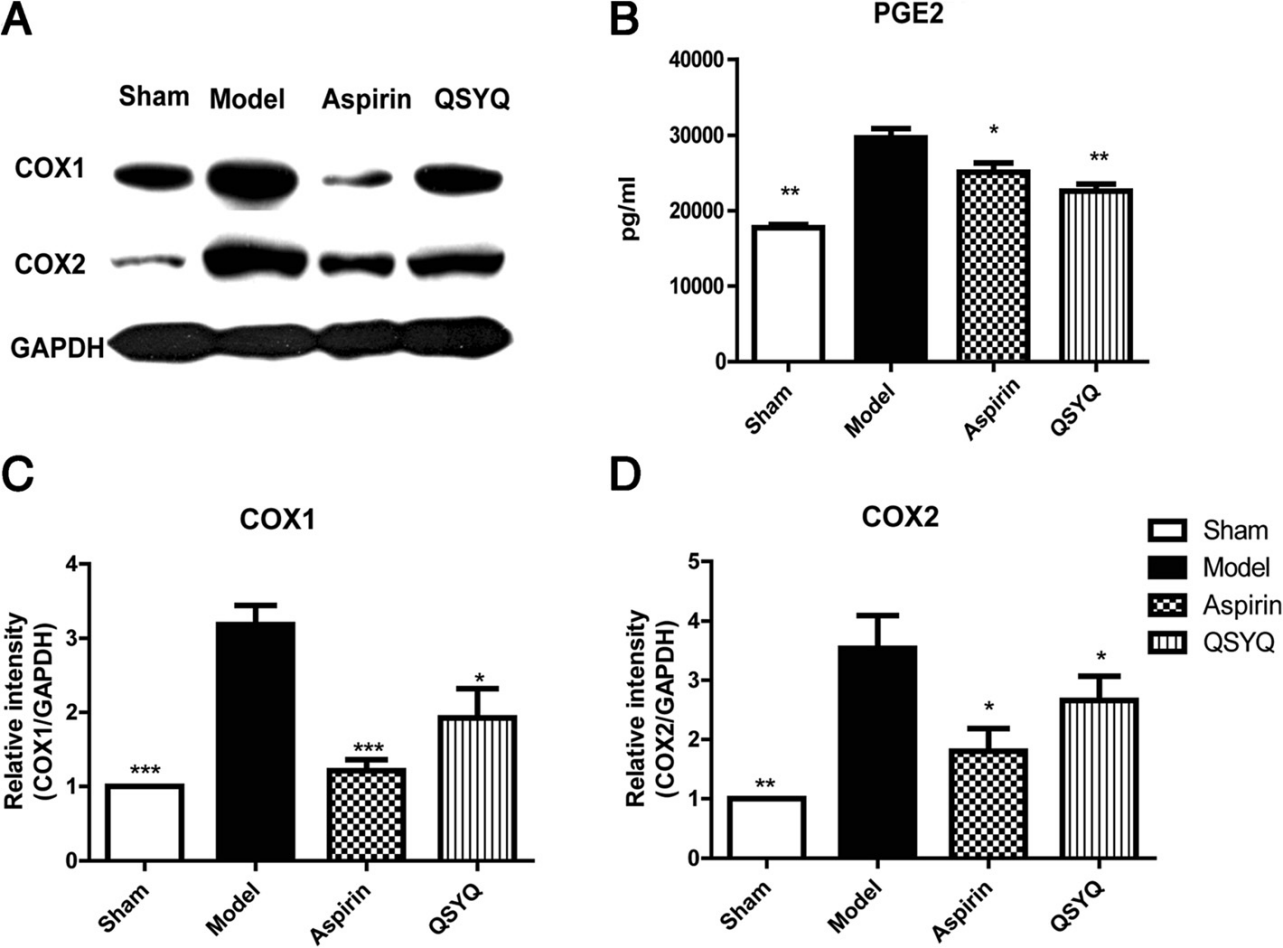
QSYQ inhibited expressions of COXs. **a** Western blot showed that expressions of COX1 and COX2 in cardiac tissues were significantly up-regulated in the model group compared with those in the sham group. In the aspirin and QSYQ-treated groups, expressions of COX1 and COX2 were all down-regulated compared with those in the model group. The inhibitory effect of QSYQ on COXs was milder than that of aspirin. **b** Expression of cardiac PGE2 in the model heart tissues was up-regulated as compared with that in the sham group and treatments with aspirin and QSYQ down-regulated expression of PGE2 towards basal level. Figure (**c**) and (**d**) were the relative quantitative illustrations of expressions of COX1 and COX2 in different groups (*P < 0.05, **P < 0.01, ***P < 0.001, other groups vs. model group, *n* = 8)

COX1 and COX2 are the rate-limiting enzymes in the AA pathway and they are activated by inflammatory cytokines. Upon activation, COXs converts AA to PGE2, which will directly induce the apoptosis and inflammatory response. Western-blot analysis showed that COX1 expression in the model group significantly increased as compared with that in the sham group. After treatment with QSYQ for 28 days, COX1 level was reduced in the QSYQ group as compared with that in the model group. The same effects on COX2 were also observed (Fig. 7).

### QSYQ inhibited PGE2 receptors, especially the subtypes of EP2/4

**Fig. 8:**
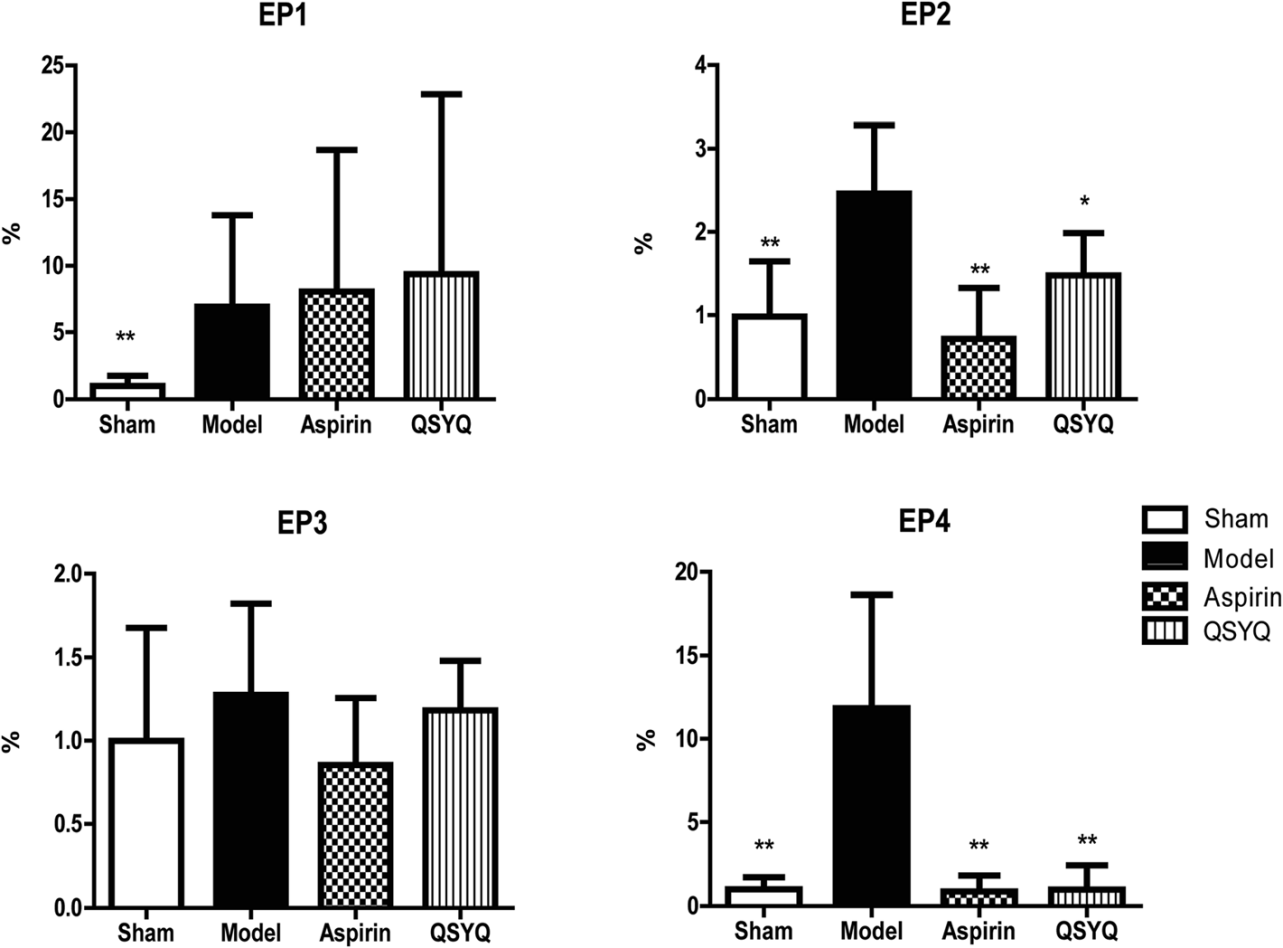
RT-PCR results of EP1/2/3/4 receptors in different groups. The results showed that in the model group, mRNA expressions of EP2/4 significantly increased compared with those in sham group. In aspirin and QSYQ groups, their expressions were restored to basal levels (*P < 0.05, **P < 0.01, other groups vs. model group, *n* = 8)

EP1to EP4 are the four subtypes of PGE2 receptors. RT-PCR results showed that mRNA expressions of EP2 and EP4 were significantly up-regulated in the model group compared with those in the sham group. QSYQ could effectively restore mRNA expressions of EP2 and EP4 towards basal levels. The effect of QSYQ on EP4 was more remarkable (Fig. 8). These results suggested that QSYQ could inhibit apoptosis by reducing EP2 and EP4 expressions.

## Discussion

Heart failure is the ultimate consequence of a large number of cardiovascular diseases. HF remains the major cause of mortality and morbidity worldwide, despite major advances in treatment approaches. The high rates of mortality and morbidity mandate discoveries of novel therapeutic strategies for it [39]. QSYQ is a patent formula of TCM and has been prescribed for the treatment of HF for many years [33]. However, the mechanism of the therapeutic effect of QSYQ remains unclear. As inflammation and apoptosis play important roles in the pathogenesis of HF, we aimed to investigate whether QSYQ could regulate these two processes in the HF rat model.

The main findings of this study showed that QSYQ may exert cardio-protective effects by regulating COXs-PGE2-P53/FasL pathway. Studies have shown that high levels of serum inflammatory markers, such as TNF-α and IL-6, were correlated with severity and prognosis of patients with HF [40, 41]. Such inflammation markers can activate the AA metabolism pathway [8], which plays an important role in the development of HF [42]. Under normal circumstances, AA resides in the cell membrane as the constituent of membrane phospholipids. When exposed to various stimuli (such as inflammation), cell membrane releases a large amount of AA from the phospholipid pools. AA can be converted to biologically active metabolites, and this process is catalyzed by enzymes COX1 and COX2. COX1 is constitutively expressed and performs physiological functions, whereas COX2 is induced by inflammatory stimuli [43]. The major metabolites, such as PGD2 and PGE2, contribute to pro-myocardial fibrosis occurring in the left ventricle. Fibrosis will reduce cardiac compliances, induce apoptosis and ultimately lead to HF [11].

In addition to inflammation, myocardial apoptosis also contributes to the progression of HF [11]. The apoptotic pathway can be divided into caspase-dependent and caspase-independent ones. The former includes both extrinsic and intrinsic apoptosis pathways. The P53-mediated intrinsic myocardial apoptosis is considered to be an important feature in HF [12]. P53 is an important regulator in cell proliferation, apoptosis and DNA repair processes in ventricular remodeling and HF [44]. Expression of P53 could be down-regulated by MDM2, which combines with P53 gene promoter and inhibits its transcription. Moreover, P53 can activate the extrinsic apoptotic pathway by inducing the expression of FasL [13]. Fas is a transmembrane protein and belongs to TNF receptor family, which mediates extrinsic apoptosis pathway.

Persistent COXs activation is also able to promote myocardial apoptosis. Prostanoids, generated by COX1, contribute to cardiac cell loss by inducing myocardial apoptosis in myocardial infarction tissues [38]. For example, PGE2 could induce apoptosis by increasing p53 gene transcriptional activity and expression of FasL. Combination of EP2/EP4 receptors with PGE2 can also promote apoptotic pathway by activating expressions of both caspases eight and nine [18]. COX2 has also been reported to induce apoptosis by inflammatory mechanisms. In the development of HF, COX2 promotes myocardial apoptosis and impairs cardiac function, which induces further secretion of COX2. This vicious cycle causes a change in heart structure as well as hemodynamic abnormalities, leading to irreversible deterioration of heart function [45].

Accumulating evidence suggests that ischemia and hypoxia, coupled with activation of various inflammatory and apoptosis pathways, play a pivotal role in HF. We studied the effects of QSYQ in the AMI-induced HF rat model and several of the findings were particularly interesting. Our research evaluated the effects of QSYQ on myocardial dysfunction, inflammation, and apoptotic pathways in the HF rat model. Ultrasonic testing after 28 days of surgery showed that values of EF and FS in the model group were significantly reduced compared with those in the sham group, indicating that HF model was successfully induced. Rats in the model group were characterized by declined diastolic and systolic myocardial performance. Our results also demonstrated that inflammatory markers, such as TNF-α and IL-6, were up-regulated in the model group. The AA-mediated apoptotic pathway was thus activated by those inflammatory factors. COX1 and COX2 are the key enzymes in the AA pathway and the levels of these two enzymes were significantly increased. COXs convert AA to PGE2 and the binding of PGE2 with EP2/4 receptors actives expression of P53 and FasL, thereby initiating apoptosis process. Our results showed that levels of EP2, EP4, P53 and FasL were all increased in the model group, indicating that the apoptotic pathway was activated in rats with HF.

After administration of QSYQ for 28 days, we found that value of ejection fraction at 28 days after surgery was increased. FS was also up-regulated by QSYQ treatment, though the difference was not statistically significant. QSYQ could also increase arterial pressure to basal level in QSYQ-treated rats. The cardiac-protective effects of QSYQ were further manifested by improved values of Max dP/dt and Min dP/dt, which are indicators of systolic and diastolic capacities. These results suggested that QSYQ can improve cardiac function in HF model rats. In the molecular level, expression of MDM2 was increased and expression of P53 was reduced in QSYQ group, compared with those in the model group. Down-regulation of P53 in QSYQ group may be mediated by increased expression of MDM2, as MDM2 is a direct inhibitor of P53 transcription. Expression of FasL in QSYQ group was also restored to normal levels, suggesting that QSYQ has anti-apoptotic effect. Furthermore, QSYQ attenuated inflammation by down-regulating expressions of TNF-α, IL-6 and COXs. The anti-inflammatory effect of QSYQ is also mediated by down-regulating EP2 and EP4, which are the receptors of PGE2. Aspirin was applied as a positive control drug in our study and showed similar effect as QSYQ. Interestingly, compared with Aspirin, QSYQ had milder inhibitory effects on both COX1 and COX2. Inhibition of COX1 is considered to be the major cause of the side-effects of Aspirin [46]. Therefore, treatment with QSYQ might be an ideal alternative for patients who develop adverse reactions when treated with Aspirin. On the whole, QSYQ is a safer medicine with fewer side effects [33].

## Conclusions

**Fig. 9:**
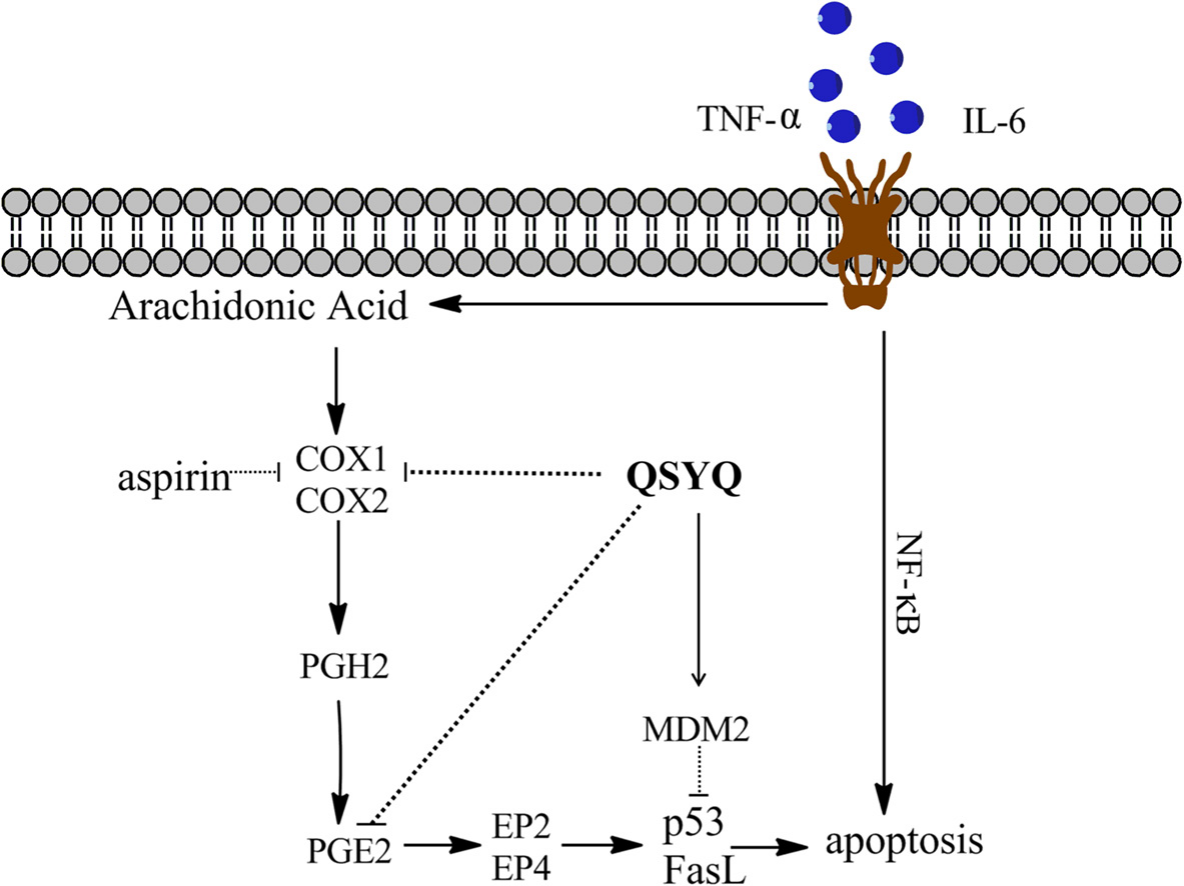
Potential mechanism of QSYQ efficacy. Treatment with QSYQ could attenuate inflammation and apoptosis in LAD-induced HF rats. One of the underlying therapeutic mechanisms of QSYQ is that it inhibits AA and P53/FasL pathway in a synergistic way

In summary, administration of QSYQ could attenuate inflammation and apoptosis in LAD-induced HF rats. One of the probable underlying therapeutic mechanisms of QSYQ is that it inhibits AA and P53/FasL pathway in a synergistic way. The simplified mechanism is shown in Fig. 9. Our study provides experimental evidence for the beneficial effects of QSYQ in the clinical application for HF.

## Additional files

Additional file 1:
**The fingerprints of QSYQ established by the HPLC-IT-TOF method.** (DOCX 172 kb) Additional file 2:
**The  primary data of echocardiographic assessment of left ventricular function.** (XLSX 12 kb)

## References

[1] Dickinson O, Chen LY, Francis GS (2014). Atrial fibrillation and heart failure: intersecting populations, morbidities, and mortality. Heart Fail Rev.

[2] Oikonomou E, Tousoulis D, Siasos G, Zaromitidou M, Papavassiliou AG, Stefanadis C (2011). The role of inflammation in heart failure: new therapeutic approaches. Hellenic J Cardiol.

[3] Anker SD, von Haehling S (2004). Inflammatory mediators in chronic heart failure: an overview. Heart.

[4] Tabas I, Glass CK (2013). Anti-inflammatory therapy in chronic disease: challenges and opportunities. Science.

[5] Seta Y, Shan K, Bozkurt B, Oral H, Mann DL (1996). Basic mechanisms in heart failure: the cytokine hypothesis. J Card Fail.

[6] Damas JK, Gullestad L, Aukrust P (2001). Cytokines as new treatment targets in chronic heart failure. Curr control trials in cardiovasc med.

[7] Levick SP, Loch DC, Taylor SM, Janicki JS (2007). Arachidonic acid metabolism as a potential mediator of cardiac fibrosis associated with inflammation. J Immunol.

[8] Wang Y, Li C, Liu Z, Shi T, Wang Q, Li D (2014). DanQi Pill protects against heart failure through the arachidonic acid metabolism pathway by attenuating different cyclooxygenases and leukotrienes B4. BMC Complement Altern Med.

[9] Force USPST (2009). Aspirin for the prevention of cardiovascular disease: U.S. Preventive Services Task Force recommendation statement. Ann Intern Med.

[10] Sohn HY, Krotz F (2006). Cyclooxygenase inhibition and atherothrombosis. Curr Drug Targets.

[11] van Empel VP, Bertrand AT, Hofstra L, Crijns HJ, Doevendans PA, De Windt LJ (2005). Myocyte apoptosis in heart failure. Cardiovasc Res.

[12] Liu P, Xu B, Cavalieri TA, Hock CE (2006). Pifithrin-alpha attenuates p53-mediated apoptosis and improves cardiac function in response to myocardial ischemia/reperfusion in aged rats. Shock.

[13] Haupt S, Berger M, Goldberg Z, Haupt Y (2003). Apoptosis - the p53 network. J Cell Sci.

[14] Tanaka M, Ito H, Adachi S, Akimoto H, Nishikawa T, Kasajima T (1994). Hypoxia induces apoptosis with enhanced expression of Fas antigen messenger RNA in cultured neonatal rat cardiomyocytes. Circ Res.

[15] Jayaraman P, Sada-Ovalle I, Nishimura T, Anderson AC, Kuchroo VK, Remold HG (2013). IL-1beta promotes antimicrobial immunity in macrophages by regulating TNFR signaling and caspase-3 activation. J Immunol.

[16] Nakamura T, Ueda Y, Juan Y, Katsuda S, Takahashi H, Koh E (2000). Fas-mediated apoptosis in adriamycin-induced cardiomyopathy in rats: In vivo study. Circulation.

[17] Vassilev LT, Vu BT, Graves B, Carvajal D, Podlaski F, Filipovic Z (2004). In vivo activation of the p53 pathway by small-molecule antagonists of MDM2. Science.

[18] Takadera T, Yumoto H, Tozuka Y, Ohyashiki T (2002). Prostaglandin E(2) induces caspase-dependent apoptosis in rat cortical cells. Neurosci Lett.

[19] Takadera T, Shiraishi Y, Ohyashiki T (2004). Prostaglandin E2 induced caspase-dependent apoptosis possibly through activation of EP2 receptors in cultured hippocampal neurons. Neurochem Int.

[20] Haque S, Yan XJ, Rosen L, McCormick S, Chiorazzi N, Mongini PK (2014). Effects of prostaglandin E2 on p53 mRNA transcription and p53 mutagenesis during T-cell-independent human B-cell clonal expansion. FASEB J.

[21] Huang SK, White ES, Wettlaufer SH, Grifka H, Hogaboam CM, Thannickal VJ (2009). Prostaglandin E(2) induces fibroblast apoptosis by modulating multiple survival pathways. FASEB J.

[22] Nakanishi M, Rosenberg DW (2013). Multifaceted roles of PGE2 in inflammation and cancer. Semin Immunopathol.

[23] Tang HY, Shih A, Cao HJ, Davis FB, Davis PJ, Lin HY (2006). Resveratrol-induced cyclooxygenase-2 facilitates p53-dependent apoptosis in human breast cancer cells. Mol Cancer Ther.

[24] Abbate A, Santini D, Biondi-Zoccai GG, Scarpa S, Vasaturo F, Liuzzo G (2004). Cyclo-oxygenase-2 (COX-2) expression at the site of recent myocardial infarction: friend or foe?. Heart.

[25] Aneja A, Farkouh ME (2008). Adverse cardiovascular effects of NSAIDs: driven by blood pressure, or edema?. Ther Adv Cardiovasc Dis.

[26] Du R-TR Y (2010). The occurrence and prevention of cyclooxygenase inhibitors and cardiovascular events. J Int Pharm Res.

[27] Ruan CH, So SP, Ruan KH (2011). Inducible COX-2 dominates over COX-1 in prostacyclin biosynthesis: mechanisms of COX-2 inhibitor risk to heart disease. Life Sci.

[28] Li X, Zhang J, Huang J, Ma A, Yang J, Li W (2013). A multicenter, randomized, double-blind, parallel-group, placebo-controlled study of the effects of qili qiangxin capsules in patients with chronic heart failure. J Am Coll Cardiol.

[29] Zhang Y, Shi P, Yao H, Shao Q, Fan X (2012). Metabolite profiling and pharmacokinetics of herbal compounds following oral administration of a cardiovascular multi-herb medicine (Qishen yiqi pills) in rats. Curr Drug Metab.

[30] Lv S, Wu M, Li M, Wang Q, Wang X, Xu L (2015). Effect of QiShenYiQi Pill on Myocardial Collagen Metabolism in Rats with Partial Abdominal Aortic Coarctation. Evid based Complement Altern Med : eCAM.

[31] Chen YY, Li Q, Pan CS, Yan L, Fan JY, He K (2015). QiShenYiQi Pills, a compound in Chinese medicine, protects against pressure overload-induced cardiac hypertrophy through a multi-component and multi-target mode. Sci rep.

[32] Hou YZ, Wang S, Zhao ZQ, Wang XL, Li B, Soh SB (2013). Clinical assessment of complementary treatment with Qishen Yiqi dripping pills on ischemic heart failure: study protocol for a randomized, double-blind, multicenter, placebo-controlled trial (CACT-IHF). Trials.

[33] Shang H, Zhang J, Yao C, Liu B, Gao X, Ren M (2013). Qi-shen-yi-qi dripping pills for the secondary prevention of myocardial infarction: a randomised clinical trial. Evid based Complement Altern Med : eCAM.

[34] Wang Y, Li C, Ouyang Y, Yu J, Guo S, Liu Z (2012). Cardioprotective Effects of Qishenyiqi Mediated by Angiotensin II Type 1 Receptor Blockade and Enhancing Angiotensin-Converting Enzyme 2. Evid based Complement Altern Med : eCAM.

[35] DW Z m, Liu C, Zhang J, Feng L, Wei Y, Liu Y (2013). Time course study of QSYQ pill acting on the myocardial inflammation in AMI rats. Lishizhen med and Chin Med.

[36] Li C, Wang Y, Qiu Q, Shi T, Wu Y, Han J (2014). Qishenyiqi protects ligation-induced left ventricular remodeling by attenuating inflammation and fibrosis via STAT3 and NF-kappaB signaling pathway. PLoS One.

[37] Li X, Wu L, Liu W, Jin Y, Chen Q, Wang L (2014). A network pharmacology study of Chinese medicine QiShenYiQi to reveal its underlying multi-compound, multi-target, multi-pathway mode of action. PLoS One.

[38] Qiu H, Liu JY, Wei D, Li N, Yamoah EN, Hammock BD (2012). Cardiac-generated prostanoids mediate cardiac myocyte apoptosis after myocardial ischaemia. Cardiovasc Res.

[39] Gutterman DD (2009). Silent myocardial ischemia. Circ J.

[40] Deswal A, Petersen NJ, Feldman AM, Young JB, White BG, Mann DL (2001). Cytokines and cytokine receptors in advanced heart failure: an analysis of the cytokine database from the Vesnarinone trial (VEST). Circulation.

[41] Chin BS, Blann AD, Gibbs CR, Chung NA, Conway DG, Lip GY (2003). Prognostic value of interleukin-6, plasma viscosity, fibrinogen, von Willebrand factor, tissue factor and vascular endothelial growth factor levels in congestive heart failure. Eur J Clin Investig.

[42] Bosetti F (2007). Arachidonic acid metabolism in brain physiology and pathology: lessons from genetically altered mouse models. J Neurochem.

[43] Arumugam TV, Arnold N, Proctor LM, Newman M, Reid RC, Hansford KA (2003). Comparative protection against rat intestinal reperfusion injury by a new inhibitor of sPLA2, COX-1 and COX-2 selective inhibitors, and an LTC4 receptor antagonist. Br J Pharmacol.

[44] Altin SE, Schulze PC (2011). p53-upregulated modulator of apoptosis (PUMA): a novel proapoptotic molecule in the failing heart. Circulation.

[45] Xu J-R (2012). study of the expression of COX-2 in the PVN and myocardial apoptosis of heart failure.

[46] Terracciano S, Aquino M, Rodriquez M, Monti MC, Casapullo A, Riccio R (2006). Chemistry and biology of anti-inflammatory marine natural products: molecules interfering with cyclooxygenase, NF-kappaB and other unidentified targets. Curr Med Chem.

