# Supplementary material for: Mechanism of QSYQ on anti-apoptosis mediated by different subtypes of cyclooxygenase in AMI induced heart failure rats
Source: BMC Complement Altern Med. 2015 Oct 7;15:352. doi: 10.1186/s12906-015-0869-z (PMC4597456; doi:10.1186/s12906-015-0869-z)
Supplement: Additional file 1: — The fingerprints of QSYQ established by the HPLC-IT-TOF method. (DOCX 172 kb) [file 12906_2015_869_MOESM1_ESM.docx]

MS-grade acetonitrile is product of Fisher Scientific (FairLawn, NJ, USA). Formic acid is product of Sigma Aldrich (St. Louis, MO, USA). Ultrapure water was prepared in our own laboratory by Milli-Q plus System (Millipore, Bedford, MA, USA). Analytical-grade solvents used for sample preparation are products of Beijing Chemical Factory (Beijing, China).

**Sample preparation for analysis**

QSYQ was weighed accurately (0.2 g) and placed into a 25 mL flask containing 10 mL 3% acetonitrile. Ultrasonic extraction (ultrasonic cleaner, 100 W, Fisher Scientific, USA) was then performed at room temperature for 5 min. The resulting mixture was filtered through a 0.22 μm membrane.

**HPLC-IT-TOF-MS^n^ conditions for chemical profile**

The HPLC-MS analysis was carried out on a Shimadzu HPLC-IT-TOF mass spectrometer equipped with an electrospray ionization (ESI) source. The chromatographic separation was performed on a Zorbax SB C_18_ column (250 × 4.6 mm, 5 μm, Agilent) at 35 ºC. Acetonitrile (A)–0.2% formic acid (B) were used as the mobile phase for analysis. The flow rate was set at 1.0 mL/min. The elution condition was applied with a gradient program as follows: 0–10 min, 3-10% A; 10–12 min, 10–15% A; 12–35 min, 15–35% A; 35–40 min, 35–40% A; 40–50 min, 40–60% A. The optimized MS operating conditions were as follows: positive and negative mode; nebulizer gas (N_2_) flow, 1.5 L/min; CDL temperature, 200 ºC; heat block temperature, 200 ºC; detector voltage, 1.60 kV; interface voltage (+), 4.5 kV; Ion accumulated time, 10 ms; repeat times, 1; collision energy was set at 50% both for MS^2^, and MS^3^; pressure of ion trap, 1.9e-002 Pa; pressure of TOF region, 1.2e-004 Pa; scan range m/z 100–1000; precursor ion isolation, 3.0000 Da. An automatic scan mode was used in the HPLC-ESI-MS^n^ analysis. The HPLC effluent was introduced into the ESI source in a post-column splitting ratio of 1:4. Aliquots of 10 μL were injected into HPLC-MS system for analysis. The typical chromatograms are shown in Fig. 1

B

A

Fig 1. HPLC-DAD-MS^n^ analysis of QSYQ. (A) HPLC-DAD chromatogram of QSYQ at 260 nm; (B) the ESI-MS/MS total ion current chromatogram of QSYQ in positive mode; (C) the ESI-MS/MS total ion current chromatogram of QSYQ negative mode.

C

B
